# Editorial: Recent Advances on Myocardium Physiology

**DOI:** 10.3389/fphys.2021.697852

**Published:** 2021-05-26

**Authors:** Norio Fukuda, Henk Granzier, Shin'ichi Ishiwata, Sachio Morimoto

**Affiliations:** ^1^Department of Cell Physiology, The Jikei University School of Medicine, Tokyo, Japan; ^2^Department of Cellular and Molecular Medicine, University of Arizona, Tucson, AZ, United States; ^3^Department of Physics, Faculty of Science and Engineering, Waseda University, Tokyo, Japan; ^4^Department of Health Sciences at Fukuoka, International University of Health and Welfare, Fukuoka, Japan

**Keywords:** heart, cardiomyopathy, calcium, muscle, sarcomere, titin (connectin), troponin

Myocardial activity reflects a multitude of signals that regulate the ability of cardiomyocytes to produce active and passive force. These mechanical activities originate in the sarcomeres highly ordered structures, composed of three filaments [thick and thin filaments, and the giant elastic protein titin (connectin)]. Early on, sarcomeres were thought to have little or no role in the regulation of the heart's pump function beyond being active force producers, in either the “*on*” or “*off* ” state, simply responding to a change in the intracellular Ca^2+^ concentration [[Ca^2+^]_i_]. Since the turn of the 21st century, cardiac researchers have applied new molecular biological and optical technologies for elucidating the detailed mechanisms of myocardial contraction. Employing these new technologies revealed that the sarcomeres are much more complex than simple “*on-off* ” machines; they are actively involved in the processes regulating the dynamics, growth, and remodeling of the heart. Likewise, these new technologies have provided new prospects to the diagnosis and treatment of heart disease. This Research Topic in *Frontiers in Physiology* is a collection of 16 original research and review papers on the physiology and pathophysiology of the myocardium, which provide compelling evidence of the rapid evolution in the field, and in particular on the roles of sarcomere proteins in health and disease.

Five of the 16 papers focus on titin, demonstrating that titin is a “hot topic” in this area of research. Before introducing each published paper in this Research Topic, we briefly summarize the current general knowledge of titin. Within the physiological sarcomere length (SL) range, titin predominantly bears passive force in mammalian myocardium (e.g., Granzier and Labeit, 2004; Fukuda et al., 2008). Titin comprises two portions: (1) the A-band portion is composed of immunoglobulin (Ig)-like and fibronectin type 3 repeats and binds to the rod segment of myosin and myosin-binding protein C (MyBP-C), and (2) the I-band portion which is the basis of viscoelasticity, has a complex sequence with distinct extensible segments: the tandem Ig segments, the PEVK segment and the segment that has a unique amino acid sequence (N2B or N2A, or both). Within the normal physiological SL range, the PEVK and unique sequences are stretched in response to SL elongation, resulting in the production of passive force. In the myocardium, the titin transcripts i.e., stiff N2B titin (containing the N2B segment) and compliant N2BA titin (containing both N2B and N2A segments) are processed differently. In the ventricle of large animals, including humans, both titin isoforms are similarly expressed, whereas N2B titin is predominantly expressed in the ventricle of small animals (e.g., small rodents).

Tharp et al. provide an informative review on titin, focusing especially on its role in dilated cardiomyopathy (DCM). DCM, a leading cause of heart failure which frequently results in sudden cardiac death, is inherited in ~50% of cases; the most frequent genetic defects are associated with truncation variants of the titin gene (*TTN*). Tharp et al. discuss the role of *TTN* mutations in the development of DCM, differential expressions of titin isoforms relating to DCM pathophysiology, and the effects of post-translational modifications of titin on cardiomyocyte functions. Future studies should be directed to elucidate whether missense variants are specifically associated with various types of cardiac disease, and the mechanism by which transcriptional and post-translational modifications contribute to DCM. As suggested by Tharp et al., altered forms of *TTN* can represent potential therapeutic targets for genetic and acquired heart disease.

Titin can be phosphorylated by various kinases. In particular, the N2B segment has physiologically important phosphorylation events mediated by various kinases, such as protein kinase A (PKA), protein kinase G (PKG), extracellular signal-regulated kinase 2 (ERK2), and calcium/calmodulin-dependent protein kinase type II delta (CaMKIIδ). Phosphorylation of the N2B segment results in a decrease in titin-based passive force, via, presumably, lengthening of the segment and as a result the whole molecule. Conversely, phosphorylation of the PEVK segment by protein kinase C alpha (PKCα) increases passive force. Although relatively lesser known to researchers in the field, recent studies have shown that protein kinase D (PKD) plays crucial roles in regulating contraction, hypertrophy and remodeling in the heart. Herwig et al. performed experiments using specimens from mice and humans, and reported that titin is a substrate of PKD, and titin-based passive force is decreased following PKD-dependent phosphorylation. Phosphosites were found in titin's various segments, including the proximal Ig region, the N2B segment and the PEVK segment. Lengthening of either the N2B segment or the PEVK segment, or both, likely underlies the PKD-dependent passive force reduction.

A sedentary lifestyle is related to increased cardiovascular risk factors and reduced ventricular wall compliance, as compared to an exercise-rich lifestyle. Chung et al. showed in rats that sedentary conditions are associated with a reduction in the content of the N2BA isoform relative to the N2B isoform in the ventricle, with no significant change in the total phosphorylation level. Likewise, the gene expression of a titin mRNA splicing factor, RNA Binding Motif 20 protein, was negatively correlated with the N2BA/N2B isoform ratio. It is therefore suggested that a sedentary lifestyle in humans reduces the N2BA/N2B isoform ratio, thereby reducing ventricular compliance. Because reduced ventricular wall compliance results in a decrease in ventricular filling and the ensuing reduction in cardiac output, the findings by Chung et al. confirm the importance of exercise in daily life activities.

Although other regions in the I-band portion of titin have been widely studied, its N2A segment, which assembles a signalosome with cardiac ankyrin repeat protein (CARP), has attained lesser attention. Lanzicher et al. investigated the effects of PKA-dependent phosphorylation and CARP on the N2A segment. They found that the N2A segment was phosphorylated by PKA, the phosphorylation did not alter the mechanical properties of the N2A segment, and that CARP blocked phosphorylation. A PKA phosphosite was revealed at the border between the N2A segment and immunoglobulin domain I81. Based on these findings, they proposed that the compliance of the N2A segment has local effects on the binding of signaling molecules and that it contributes to strain- and phosphorylation-dependent mechano-signaling.

Titin can be a therapeutic target in the treatment of heart disease associated with stiffening of ventricular muscle. Kolijn et al. provided evidence showing that nitric oxide-independent activation of soluble guanylyl cyclase (sGC) reduces myocyte passive force from the ventricles of rats and humans by improving the activities of PKA and PKG. They studied the effects of nitric oxide-independent activation of sGC on cardiomyocyte function in a hypertensive rat model with diastolic dysfunction and in biopsies from human heart failure with preserved ejection fraction (HFpEF). Passive force was increased in cardiomyocytes from Dahl salt-sensitive rats as well as in those from human myocardial biopsies. The increase in passive force was related to hypophosphorylation of the total titin and to deranged site-specific phosphorylation of titin's elastic segments, accompanied by reduced levels of PKG and PKA activity, along with dysregulation of hypertrophic pathway markers such as CaMKII, PKC, and ERK2. It was shown that sGC activator improved cardiomyocyte function, reduced inflammation and oxidative stress, improved sGC-PKG signaling and normalized hypertrophic kinases, suggesting that it is a potential treatment option for HFpEF patients and, presumably, for cases with increased hypertrophic signaling.

Hypertrophic cardiomyopathy (HCM) is a genetic disorder caused by mutations in different genes but mainly encoding myofilament proteins. While mutations in sarcomere proteins were discovered ~30 years ago, the cellular and molecular mechanisms for the development of HCM remain unclarified. Many efforts have been made to treat HCM in the clinical setting; however, they have largely been unsuccessful, and no curative treatment has been developed. Chowdhury et al. shed light on this issue by providing important evidence that acceleration of the sarcoplasmic reticulum function prevents progression of cardiac troponin (Tn) T (cTnT)-linked HCM. They investigated a mouse model expressing the mutant cTnT-R92Q (which is linked to HCM and induces an increase in myofilament Ca^2+^ sensitivity and diastolic dysfunction), and they found that early amelioration of the diastolic dysfunction via enhanced [Ca^2+^]_i_ reduction by phospholamban knockout prevented the development of the HCM phenotype in this mouse model.

Regulation of cardiac physiology occurs via kinases that reversibly phosphorylate ion channels, Ca^2+^ handling machinery and signaling effectors. Essandoh et al. reviewed zDHHC enzymes and substrate S-acylation in myocardium physiology. Palmitoylation or S-acylation, the post-translational modification of cysteines with saturated fatty acids, plays roles in regulating various functions of proteins in cardiomyocytes. S-acylation is catalyzed by the zDHHC family of S-acyl transferases that localize to intracellular organelle membranes or the sarcolemma. Recent works have revealed the functions of S-acylation in the heart, particularly in the regulation of cardiac excitation-contraction coupling. Although the dynamics and functions of these modifications in myocardium physiology have not been interrogated, proteins with critical signaling roles in the heart are likewise S-acylated, including receptors and G-proteins. Proteomic studies to interrogate alterations in cardiac palmitoylome in models with manipulated zDHHC enzyme activities will facilitate the discovery of novel molecular mechanisms giving rise to cardiac disease.

Four papers have added to the recent advances in the understanding of cardiomyopathies. Copeland et al. investigated whether TnI and MyBP-C phosphorylation levels are decreased in myocardium from non-HCM patients with aortic stenosis. Previous studies indicated that phosphorylation levels of these proteins are extremely low in patients with hypertrophic obstructive cardiomyopathy (HOCM), an obstructive variant of HCM with aortic stenosis, compared to in patients with non-obstructive HCM. They found that the phosphorylation levels of TnI and MyBP-C were significantly lower in aortic stenosis patients' hearts than in donor hearts, suggesting that left ventricular pressure overload is a major factor in inducing the secondary phenotype of HOCM hearts. The mechanisms of low phosphorylation await further investigations. However, as Copeland et al. pointed out, such mechanisms are likely coupled with downregulation of β adrenoceptors and the ensuing reduction of PKA activity as well as with increased phosphatase activities.

McDonald et al. analyzed the myofibrillar basis for contractile dysfunction in failing human myocardium. They introduced the novel parameter “peak power output normalized to isometric force (PNPO),” and measured the contractile properties in cardiac myocytes from left ventricular mid-wall biopsies of donor and failing human hearts. It was found that maximal Ca^2+^-activated isometric force, maximal force development rates (*k*_*tr*_), and Ca^2+^ activation dependence of *k*_*tr*_ were similar between the groups. The Ca^2+^ activation dependence of loaded shortening and power output were then examined. PNPO was decreased by ~10% from maximal Ca^2+^ to half-maximal Ca^2+^ activations in both donor and failing groups. The SL dependence of PNPO was diminished in failing myocytes. Their findings suggest that the altered SL-dependent regulation of myofilament function impairs ventricular performance in failing human hearts.

Groen et al. compared the effects of mutations associated with HCM or DCM. In their study, the native Tn complex in skinned trabecular fibers of guinea pigs was replaced with recombinant human cardiac troponin complexes containing different human cardiac TnT (hcTnT); i.e., HCM-associated hcTnT^R130C^, DCM-associated hcTnT^Δ*K*210^ or wild type hcTnT. They found that lengthening the fibers from 1.1 slack length (*L*_0_) to 1.25 *L*_0_ increased Ca^2+^ sensitivity in fibers containing hcTnT^R130C^, did not alter Ca^2+^ sensitivity in the wild type, and decreased Ca^2+^ sensitivity in fibers containing hcTnT^Δ*K*210^. As they noted, the findings with the wild type and hcTnT^Δ*K*210^ were unexpected. As an exemplification, Mashali et al. (2021) recently reported that length-dependent activation is preserved and virtually identical in large numbers of failing and non-failing human hearts. Nevertheless, the findings by Groen et al. may be important in the primary effects of mutations on length-dependent activation that contribute to the development of the diverging phenotypes in HCM and DCM in humans.

Pioner et al. reviewed DCM associated with mutations in the Duchenne muscular dystrophy (DMD) gene encoding dystrophin. They discussed the physiological roles of dystrophin in myocardial functions in cardiac development, as well as in the progression of the disease. Dystrophin is localized at the cytoplasmic face of the muscle cell plasma membrane, or at the sarcolemma, and particularly at the components within a cytoskeletal lattice (costameres). Costameres couple the sarcolemma with the Z disk of myofibrils via interacting proteins. Recently, studies using human induced pluripotent stem cells (hiPSCs) have advanced to reveal the pathogenesis of DMD. It has been suggested that the pathogenic mechanisms appear early in the disease progression as a combination of the developmental consequences of the absence of full-length dystrophin in cardiomyocytes. Gene therapies have been emerged as a promising approach for DMD; i.e., a gene therapy with micro dystrophin, up-regulation of utrophin or exon skipping approaches are currently the most promising ways to rescue or at least mitigate the DMD phenotype.

Experimentations using living isolated single cardiomyocytes have dramatically advanced our understanding of myocardium physiology and pathophysiology. Wright et al. reported a new approach using the novel CytoCypher High-Throughput System (CC-HTS). This system allows for assessment of shortening of sarcomeres, cell length, Ca^2+^ handling and cellular morphology of nearly four cells per minute. They explored basic morphological and functional characteristics of rat, mouse, guinea pig, and human cells. Likewise, they analyzed the effects of agents that affect actomyosin interaction involved in cardiomyocyte contraction. The introduction of HTS methodology for myocytes has dramatically increased the speed of measurement and improved the statistical power while reducing the number of myocyte isolations needed for a study.

The chambers of the heart fulfill different hemodynamic functions. By taking advantage of the high-throughput contractility set-up, Nollet et al. measured the contractile function of >2,000 cultured cells from the atrium, right ventricle (RV), left ventricle (LV) and interventricular septum (IVS) of the healthy rat. They report two important findings. First, compared to ventricular cardiomyocytes, atrial cells showed a twofold lower shortening amplitude and ~1.5-fold slower kinetics of shortening and lengthening. The interventricular differences were much smaller; RV cells displayed 12–13% less fractional shortening and 5–9% slower shortening and 3–15% slower lengthening kinetics compared to LV / IVS cells. These findings are in line with the contractile differences observed at the atrioventricular level in traditional studies. Their study highlights the importance of extensive, unbiased sampling when performing studies on cardiomyocyte contractility.

Two papers review important basic characteristics of myocardial functions. Ishii et al. discussed the effects of temperature on the contractile performance of mammalian striated muscle. In myocardium, either a decrease or an increase in temperature enhances its contractility: First, a rapid decrease in solution temperature generates contraction in intact cardiac muscle, a phenomenon known as “rapid cooling contracture (RCC).” RCC is caused by Ca^2+^ release from the sarcoplasmic reticulum (SR) through ryanodine receptors. Second, rapid heating induces reversible cardiomyocyte shortening (Oyama et al., 2012). It is important that this heating-induced contraction is regulated at the sarcomere level. Ishii et al. discussed their previous work (Ishii et al., 2019) demonstrating that reconstituted cardiac thin filaments exhibit sliding movements at >~35°C in the absence of Ca^2+^. One possible mechanism for the “thermal activation” of thin filaments is partial dissociation of the Tn-tropomyosin complex from F-actin, thereby allowing the actomyosin interaction.

Myocardial cells exhibit spontaneous beating. Most notably, pacemaker cells beat at a relatively fixed frequency, and similar spontaneous beating likewise occurs in embryonic and neonatal cardiomyocytes. Ryanodine receptors (RyR) in the SR play an important role in spontaneous beating, coupled with rhythmic changes in [Ca^2+^]_i_. Cohen and Safran focused on the role of RyR channels and SR Ca^2+^ pump functions in the cytoplasmic [Ca^2+^] cycles, and constructed a minimal mathematical model based on the Van-der-Pol equation. Accordingly, the dynamics of spontaneous beating, as well as paced isolated cardiomyocytes, were simulated as derived from Ca^2+^ pumps and channels dynamics. Spontaneous contraction-relaxation cycles in normal ventricular myocytes may result in fatal arrythmias. Therefore, understanding not only the biology but also the physics behind cardiomyocyte beating can facilitate the design of better treatments for arrhythmias.

Washio et al. add a mathematical simulation paper based on the bio-hierarchy of the heart, i.e., sarcomeres, myofibrils, cells, muscle fibers and the organ. Namely, by taking into account the fiber reorientation in the heart, they refined their UT-Heart model (e.g., Sugiura et al., 2012). From the viewpoint of myocardium physiology, they provided three important findings. First, the force-velocity relationship is crucial in transferring the fiber shortening strain to active force in myofiber reorientation. Second, the algorithm developed in their study facilitates assessment of homogeneity in active force and fiber shortening strain, and results in near-optimal pumping performance. Third, the reorientation mechanism may degrade the heart's pumping performance if myocardial contractility becomes inhomogeneous. Future studies are warranted to investigate the effects of changes in the dynamics of a component in the heart's bio-hierarchy on its physiological and pathophysiological functions.

This Research Topic that although many challenges remain, considerable progress has been made in understanding the physiology and pathophysiology of the heart. As revealed by the authors, by taking advantage of current technologies as well as developing new techniques and approaches, obstacles toward elucidating the physiology and pathophysiology of the heart can be overcome, bringing us closer toward understanding and ultimately treating disorders of the heart.

## Author Contributions

All authors listed have made a substantial, direct and intellectual contribution to the work, and approved it for publication.

## Conflict of Interest

The authors declare that the research was conducted in the absence of any commercial or financial relationships that could be construed as a potential conflict of interest.

## Abbreviations

[Ca^2+^]_i_: intracellular Ca^2+^ concentration
CaMKII: calcium/calmodulin-dependent protein kinase type II
DCM: dilated cardiomyopathy
ERK2: extracellular signal-regulated kinase 2
HCM: hypertrophied cardiomyopathy
MyBP-C: myosin-binding protein C
PKA: protein kinase A
PKC: protein kinase C
PKD: protein kinase D
PKG: protein kinase G
SL: sarcomere length
SR: sarcoplasmic reticulum
sGC: soluble guanylyl cyclase
Tn: troponin.
